# Abnormal Neural Activity in Different Frequency Bands in Parkinson’s Disease With Mild Cognitive Impairment

**DOI:** 10.3389/fnagi.2021.709998

**Published:** 2021-08-20

**Authors:** Siming Rong, Piao Zhang, Chentao He, Yan Li, Xiaohong Li, Ruitao Li, Kun Nie, Sifei Huang, Limin Wang, Lijuan Wang, Yuhu Zhang

**Affiliations:** Department of Neurology, Guangdong Neuroscience Institute, Guangdong Provincial People’s Hospital, Guangdong Academy of Medical Sciences, Guangzhou, China

**Keywords:** resting-state functional MRI, Parkinson’s disease with mild cognitive impairment, fractional amplitude of low-frequency fluctuation, degree centrality, frequency dependence

## Abstract

**Background**: Abnormal spontaneous neural activity is often found in patients with Parkinson’s disease with mild cognitive impairment (PD-MCI). However, the frequency dependence of neuronal interaction activities, especially the fractional amplitude of low-frequency fluctuation (fALFF) and degree centrality (DC), in PD-MCI is still unclear. Thus, this study aimed to explore the frequency dependence of PD-MCI based on fALFF and DC maps.

**Methods**: Twenty-four patients with PD-MCI, 42 PD patients with normal cognition (PD-NC), and 33 healthy controls (HCs) were enrolled. Neuropsychological assessments and resting-state functional MRI (rs-fMRI) were performed. The fALFF and DC values in the conventional, slow4 and slow5 frequency bands were compared among the groups.

**Results**: In the conventional frequency band, the DC value in the left precentral area was decreased in PD-MCI patients, while that in the right fusiform area was increased in PD-NC patients compared with HCs. Regarding fALFFs, both the PD-MCI and PD-NC patients had decreased values in the right precentral area compared with those of the HCs. The fALFFs did not differ between PD-MCI and PD-NC patients. The fALFF results in the slow4 subfrequency band were consistent with those in the conventional frequency band. In the slow5 band, the DC value in the left middle temporal lobe was higher in PD-MCI patients than in PD-NC patients and was positively correlated with the performance of the PD-MCI patients on the Montreal Cognitive Assessment (MoCA). Additionally, both PD-MCI and PD-NC patients showed lower fALFF values in the bilateral putamen than the HCs, and the fALFF in the bilateral putamen was negatively correlated with the Hoehn and Yahr stages of PD-MCI. The fALFF in the left putamen was negatively correlated with the scores of PD-MCI patients on the Movement Disorder Society-Unified Parkinson Disease Rating Scale Part III (MDS-UPRDS-III).

**Conclusion**: Our results suggested that abnormal neuronal activities, such as fALFF and DC, are dependent on frequency in PD-MCI. Some subfrequency bands could distinguish PD-MCI from PD. Our findings may be helpful for further revealing the frequency-dependent resting functional disruption in PD-MCI.

## Introduction

Parkinson’s disease (PD), a neurodegenerative disease characterized by typical motor symptoms such as bradykinesia, resting tremors, and rigidity, is also accompanied by nonmotor symptoms, including cognitive impairment, anxiety, depression, and sleep disorders (Postuma et al., 2015). Up to 80% of patients with PD will develop dementia in the late stage, which may substantially increase medical costs and reduce the quality of life (Aarsland et al., 2003; Lawson et al., 2016). PD with mild cognitive impairment (PD-MCI) represents the early stage of cognitive impairment, and approximately 91% of PD-MCI cases eventually progress to PD with dementia (PDD; Aarsland et al., 2017). Due to the high risk of progressing to PDD, the early detection of PD-MCI could allow timely medical intervention and delay the progression to PDD.

Resting-state functional magnetic resonance imaging (rs-fMRI) can be used to assess interregional correlations between blood oxygen level-dependent (BOLD) signal fluctuations and neuronal interaction activity and has unique advantages in clinical situations (Biswal et al., 1995; Fox and Raichle, 2007). In recent years, analytical techniques [functional connectivity (FC), independent component analysis (ICA), graph theory, etc.] for rs-fMRI data have been effectively used to characterize brain network functions in PD, especially in the cognitive impairment stage (Wolters et al., 2019), and reduced network connectivity was found to correlate with cognition in PD patients with cognitive impairment. However, several methods of characterizing the local properties of rs-fMRI signals, including the amplitude of low-frequency fluctuations (ALFFs), the fractional amplitude of low-frequency fluctuations (fALFFs), and degree centrality (DC), have not been fully used to investigate abnormal functional patterns in PD. The ALFF value represents the mean amplitude of fluctuations within the low-frequency range and directly characterizes spontaneous brain activity at each voxel (Zang et al., 2007). fALFFs are defined as the proportions of these low-frequency fluctuations (0.01–0.1 Hz) to the whole signal. DC is proposed to map the degree of intrinsic FC across the brain to reflect a stable property of cortical network architecture at the voxel level (Buckner et al., 2009). Most previous studies focused on the use of ALFF to investigate the disruption of neural activity in PD-MCI, while fewer studies have focused on fractional amplitude of low-frequency fluctuation (fALFF) and DC values. These voxelwise metrics can define different brain function characteristics in the resting state and highlight disease features. Therefore, we aimed to utilize these metrics to investigate the abnormality of neural activity in PD-MCI and to assess the clinical relationship.

In addition, most previous studies on PD analyzed neurol activity in only the conventional low-frequency band between 0.01 and 0.1 Hz (Biswal et al., 1995). To the best of our knowledge, rs-fMRI has a relatively high temporal resolution (usually 2 s) and therefore supports frequency-dependent analysis of subfrequency bands such as slow5 (0.01–0.027 Hz) and slow4 (0.027–0.073 Hz). Hou et al. (2014) conducted a frequency-dependent analysis of PD and found that ALFF abnormalities in PD patients were more robust in certain frequency bands (Hou et al., 2014). However, all of the local metrics might have frequency dependence, and more specific frequency bands need to be considered in the blood-oxygen-level-dependent (BOLD)-fMRI analysis of brain activity in PD, especially in PD-MCI. Therefore, we aimed to investigate local abnormalities in patients with PD-MCI and whether these local metrics are frequency-dependent.

## Materials and Methods

### Participants

Participants in this study were recruited from the Department of Neurology, Guangdong Neuroscience Institute, Guangdong Provincial People’s Hospital, Guangzhou, China. Sixty-six patients with PD and 33 education-, age-, and sex-matched healthy controls (HCs) were enrolled in this study. PD was clinically diagnosed according to the Queen Square Brain Bank criteria as follows: onset age of movement-related symptoms greater than 50 years, aged between 50 and 80 years, education level higher than primary school, no presence of dementia according to the Movement Disorders Society criteria (Emre et al., 2007), Hoehn and Yahr stage ≤3, and no severe depression or anxiety [Hamilton Depression Scale (HAMD) ≤35 points and Hamilton Anxiety Scale (HAMA) ≤29 points]. The medical treatments included levodopa, dopaminergic agonists, and monoamine oxidase, and catechol-O-methyltransferase inhibitors, and the levodopa equivalent daily dose (LEDD) was appropriately calculated (Tomlinson et al., 2010). Those with a medication history of acetylcholinesterase inhibitors were excluded from this study. Volunteers with no neurological or mental illnesses and no recent medication history of acetylcholinesterase inhibitors were rated by the Mini-Mental State Examination (MMSE), and those with MMSE scores higher than the Chinese cutoff value were selected as the HCs (Chen et al., 2016). All subjects provided written informed consent to participate in the study protocol, which was approved by the Medical Ethics Committee of Guangdong Provincial People’s Hospital [no. GDREC2018338H(R1)].

#### Clinical and Neuropsychological Evaluation

The motor functions of most of the subjects were evaluated by the Movement Disorder Society-sponsored revision of the Unified Parkinson’s Disease Rating Scale part III (MDS-UPDRS-III) under the “OFF” condition. The neurobehavioral assessments included the HAMA and the HAMD. Neuropsychological assessments, including the MMSE and Montreal Cognitive Assessment (MoCA), were used to determine global cognitive efficiency. The animal fluency test and Picture Arrangement (Wechsler Adult Intelligence Scale, WAIS-R) were used to assess executive functions; digit span and digit symbol tests were used to assess attention and working memory; logical memory and immediate memory tests from the Wechsler Memory Scale (WMS) and vocabulary and similarities subtests (WAIS-R) were used to assess language; block design and object assembly were used to assess visuospatial function. The patients with PD were divided into PD with normal cognition (PD-NC) and PD-MCI according to the Level II criteria of the Movement Disorder Society (MDS) Task Force guidelines (Litvan et al., 2012). The diagnosis of PD-MCI was based on the criterion of a score ≥1.5 standard deviation (SD) unit below the mean in at least two of ten tests in five domains (Gong, 1983; Gong et al., 1989).

#### Neuroimaging

##### MRI Data Acquisition and Protocol

Functional MRI data were collected from all PD patients in the morning. Brain functional MRI was performed using a 3.0 T scanner (Signa Excite HD GE Healthcare, Milwaukee, WI, USA) with an 8-channel head coil. During scanning, the subject was positioned supine in the framework of the scanner with foam padding to limit head movement and wore earplugs to reduce the impact of acoustic noise. All subjects were instructed to keep their eyes closed, to not think of anything in particular, and not fall asleep. The rs-fMRI data parameters were as follows: gradient-echo echo-planar imaging (GRE-EPI) sequence with repetition time (TR)/echo time (TE) = 2,000/30 ms, matrix = 64 × 64, 30 axial slices covering the entire brain, field of view = 240 × 240 mm, slice thickness = 4 mm, interslice space = 1 mm, NEX = 1, voxel size = 3.75 mm × 3.75 mm × 4 mm, time points = 186, and 30 axial slices covering the entire brain for a total of 5,580 images. Axial scans were acquired in parallel to the anterior-posterior commissure (AC-PC) line. High-resolution 3D T1-weighted anatomical images were obtained for coregistration with the functional data. A fast spoiled gradient recalled echo inversion recovery (FSPGRIR) sequence was used to acquire sagittal T1-weighted images with a TR/TE = 8.4/3.3 ms, matrix = 256 × 256, flip angle = 13°, slice thickness = 1 mm, and voxel size = 0.94 mm × 0.94 mm ×1 mm.

##### MRI Data Preprocessing

The resting-state fMRI data were processed by using SPM12 software^1^ and RESTplus Version 1.2^2^ on the MATLAB 2014a platform. The first 10 time points for each participant were removed to prevent scanner noise impacts. The data preprocessing procedure included slice timing, realignment, and spatial normalization. First, an individual structural image was coregistered to the mean functional image and then segmented into gray matter (GM), white matter (WM), and cerebrospinal fluid (CSF) by using “New Segment.” Diffeomorphic anatomical registration with the exponentiated Lie algebra (DARTEL) tool was used to compute the transformation from individual space to Montreal Neurological Institute (MNI) space and vice versa (resampling voxel size = 3 mm × 3 mm × 3 mm). Smoothing was performed with a 4 mm full-width half-maximum (FWHM) Gaussian kernel. After removing the linear trend, covariates, including head motion parameters (using the Friston 24-parameter) and WM and CSF signals, were regressed. The time courses were filtered to a 0.01–0.08 Hz band to reduce high-frequency noise and low-frequency drifts.

To investigate the frequency effects on the group comparisons, in addition to the conventional frequency band (0.01–0.08 Hz), the full frequency range (0–0.25 Hz) was divided into five different bands: slow6 (0–0.01 Hz), slow5 (0.01–0.027 Hz), slow4 (0.027–0.073 Hz), slow3 (0.073–0.198 Hz), and slow2 (0.198–0.25 Hz). Because the slow6, slow3 and slow2 bands were affected by very low-frequency drifting, WM signals, and high-frequency physiological noise, we chose to analyze only the subfrequency bands slow4 and slow5.

#### Local Brain Network Metrics Calculations (Fractional ALFF and DC)

Each preprocessed scan was subjected to fALFF analysis, and the fALFF calculation was based on the fast Fourier transform algorithm. Using the algorithm, each time course was converted to the frequency domain. Then, the square root of the power spectrum at each frequency was averaged across the filtered band (0.01–0.08 Hz). This averaged square root was taken as the ALFF at each voxel. Then, the fALFF was calculated from the ratio of the power spectrum of low-frequency (0.01–0.08 Hz) to that of the entire frequency range. The same method was applied to the slow4 and slow5 subfrequency bands.

The DC metric is used to assess the summed FC of each individual voxel with all voxels of the brain. After image preprocessing, bandpass filtering (a conventional low-frequency band and four subfrequency bands as described above) was performed. All pairwise Pearson correlation coefficients were calculated, and the cutoff for the correlation coefficient was set to 0.25. The weighted DC map was obtained and then spatially smoothed with a 4 × 4 × 4 mm FWHM Gaussian kernel. The DC value of each voxel was divided by the global mean DC for standardization.

#### Statistical Analysis

Statistical analysis was performed using SPSS Statistics version 25.0 (IBM SPSS Statistics, Armonk, NY, USA). The Kolmogorov-Smirnov test was used to assess normal distribution, and the normally distributed continuous variables were then compared by analysis of covariance (ANCOVA) and unpaired *t*-tests. The Kruskal-Wallis H and Mann-Whitney U tests were used to compare nonnormally distributed parameters, and the chi-square test was used to compare categorical variables.

Two sample *t*-tests were performed to compare fALFF and DC values among the PD-MCI, PD-NC, and HC groups. The T-map threshold was set by multiple comparison correction based on the Gaussian random field theory (GRF corrected, voxelwise *p* < 0.005, clusterwise *p* < 0.05, two-tailed). We further set a lower threshold to examine the results without significance (GRF corrected, voxelwise *p* < 0.01, clusterwise *p* < 0.05, two-tailed).

With the peak voxels of abnormal regions as the spherical centers, spherical regions of interest (ROIs) were constructed around these abnormal regions (6 mm radius) to extract the local metrics in the significantly abnormal regions. Correlation analysis was conducted with the RESting-state fMRI data analysis Toolkit (REST), and Pearson correlation analysis was used to assess the associations between local metrics in significant regions and clinical characteristics; *p* < 0.05 indicated statistical significance.

### Results

#### Clinical and Neuropsychological Evaluation

Among the PD-NC, PD-MCI, and HC groups, no significant differences in sex, age, or education were noted. The HAMD (*H* = 24.76, *p* < 0.001), HAMA (*H* = 18.85, *p* < 0.001), MMSE (*H* = 13.91, *p* < 0.001), and MoCA (*H* = 40.88, *p* < 0.001) scores were significantly different among the three groups. The HAMD and HAMA scores were higher in the PD-MCI and PD-NC groups than in the HC group, and the MMSE and MoCA scores of the PD-MCI group were lower than those of the PD-NC and HC groups. The duration of disease, H-Y stage, MDS-UPDRS-III scores, and LEDDs were not significantly different between the PD-NC and PD-MCI groups (Table 1).

**Table 1:** Demographic and neuropsychological information.

	PD-NC	PD-MCI	HC	Static	*p*
Subjects	42	24	33		
Male/female	26/16	17/7	17/16	2.22^b^	0.33
Age	64.48 (68.72)	65.75 (6.03)	63.33 (5.31)	1.06^a^	0.35
Education	10.83 (3.33)	9.75 (3.47)	11.39 (3.15)	4.04^c^	0.13
HAMD	8.55 (9.05)	10.08 (7.60)	2.06 (2.08)	24.76^c^	<0.001
HAMA	7.33 (7.18)	9.13 (6.93)	2.85 (2.46)	18.85^c^	<0.001
MMSE	28.24 (1.89)	26.14 (2.88)	28.58 (1.50)	13.91^c^	<0.001
MoCA	23.26 (3.32)	18.58 (4.44)	26.21 (1.90)	40.88^c^	<0.001
Duration of disease	2.87 (2.19)	2.81 (2.33)	-	−0.23^d^	0.82
LEDD	182.38 (336.56)	147.08 (241.59)	-	−0.27^d^	0.79
MDS-UPDRS-III	31.17 (13.21)	34.25 (16.82)	-	−0.75^d^	0.46
H-Y stage	2.00 (0.52)	2.08 (0.64)	-	3.52^b^	0.48

*The data are shown as the mean (standard deviation). Abbreviations: MMSE, Mini-Mental State Examination; MoCA, Montreal Cognitive Assessment; HAMA, Hamilton Anxiety Scale; HAMD, Hamilton Depression Scale; HY, Hoehn and Yahr Scale; MDS-UPDRS-III, Part III of the Movement Disorder Society-sponsored revision of the Unified Parkinson’s Disease Rating Scale, LEDD, levodopa equivalent daily dose. a: One-way analysis of variance; b: Pearson’s chi-square (*X*^2^); c: Kruskal-Wallis H test; d: Mann–Whitney *U* test*.

#### Groups Difference in the DC Map

**Figure 1:**
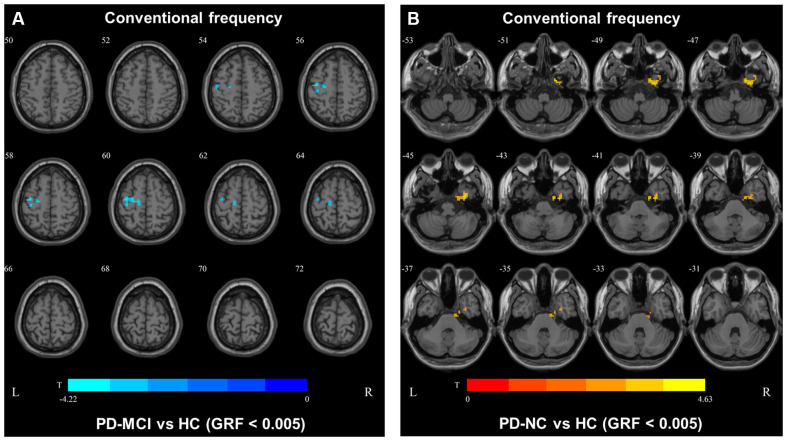
Degree centrality (DC) map differences among the groups in conventional frequency band. The DC results were compared by two sample *t*-tests (GRF corrected, voxel *p* < 0.005, cluster *p* < 0.05). The color bar on the bottom indicates the statistical t value. The warm (cold) color indicates a significantly increased (decreased) distant DC in the comparison. Additional details regarding these regions with difference are shown in Table 2. **(A)** Decreased DC in PD-MCI patients between PD-MCI and HC. **(B)** Increased DC in PD-NC patients between PD-NC and HC.

In the conventional frequency band, the DC value in the left precentral gyri was decreased in PD-MCI patients compared with HCs, and that in the right fusiform gyri was increased in PD-NCs compared with HCs. No difference in the conventional frequency band was found between the PD-NC and PD-MCI groups (Figure 1, Table 2).

**Table 2:** Brain regions showing significant degree centrality (DC) differences among the three groups.

1. Conventional frequency band								
Contrast	Cluster No.	Number of voxels	Main brain regions	Peak MNI coordinate			*t* value	*p* value
				*X*	*Y*	*Z*		
PD-MCI vs. HC	1	63	Precentral_L	−36	−9	60	−4.2247	0.005
PD-NC vs. HC	1	97	Fusiform_R	30	−9	−48	4.6319	0.005
**2. Slow4**
**Contrast**	**Cluster No.**	**Number of voxels**	**Main brain regions**	**Peak MNI coordinate**			***t* value**	***p* value**
				***X***	***Y***	***Z***		
PD-MCI vs. HC	1	112	Precentral_L	−24	−27	54	−4.3213	0.005
	2	63	Right frontal lobe/precentral gyri	30	−27	57	−4.5191	0.005
PD-NC vs. HC	1	72	Fusiform_R	33	−6	−45	4.1852	0.005
**3. Slow5**
**Contrast**	**Cluster No.**	**Number of voxels**	**Main brain regions**	**Peak MNI coordinate**			***t* value**	***p* value**
				***X***	***Y***	***Z***
PD-NC vs. PD-MCI	1	44	Left middle temporal gyri	−63	−45	−3	−4.1118	0.005

**Figure 2:**
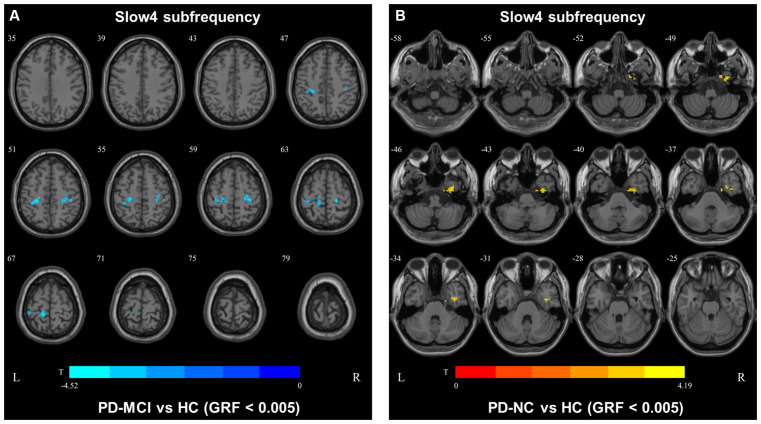
DC map differences among the groups in slow4 subfrequency band. The DC results were compared by two sample *t*-tests (GRF corrected, voxel *p* < 0.005, cluster *p* < 0.05). The color bar on the bottom indicates the statistical t value. The warm (cold) color indicates a significantly increased (decreased) distant DC in the comparison. Additional details regarding these regions with difference are shown in Table 2. **(A)** Decreased DC in PD-MCI patients between PD-MCI and HC. **(B)** Increased DC in PD-NC patients between PD-NC and HC.

**Figure 3:**
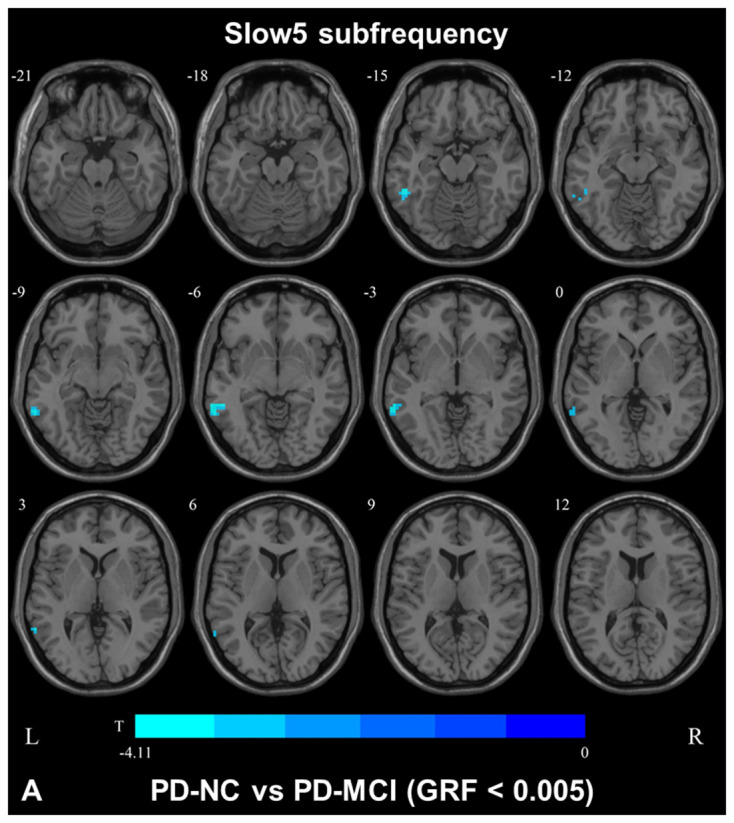
DC map differences among the groups in slow5 subfrequency band. The DC results were compared by two sample *t*-tests (GRF corrected, voxel *p* < 0.005, cluster *p* < 0.05). The color bar on the bottom indicates the statistical *t* value. The cold color indicates a significantly decreased distant DC in the comparison. Additional details regarding these regions with difference are shown in Table 2. **(A)** Decreased DC in PD-NC patients between PD-NC and PD-MCI.

In slow4, The DC value in the right precentral/postcentral area was decreased in PD-MCI patients compared with the HCs (Figure 2, Table 2). However, in slow5, the DC values in the left middle temporal gyri were decreased in the comparison of the PD-MCI and PD-NC groups, but no difference was observed when compared with the HCs (Figure 3, Table 2).

**Table 3:** Brain regions showing significant fractional amplitude of low-frequency fluctuation (fALFF) differences among the three groups.

1. Conventional frequency band								
Contrast	Cluster No.	Number of voxels	Main brain regions	Peak MNI coordinate			*t* value	*p* value
				*X*	*Y*	*Z*		
PD-MCI vs. HC	1	69	Right precentral gyri	60	−9	18	−5.3866	0.005
PD-NC vs. HC	1	56	Right precentral gyri	57	−12	24	−4.0936	0.005
**2. Slow4**
**Contrast**	**Cluster No.**	**Number of voxels**	**Main brain regions**	**Peak MNI coordinate**			***t* value**	***p* value**
				***X***	***Y***	***Z***
PD-MCI vs. HC	1	178	Right precentral gyri	60	−9	−18	−5.5194	0.005
PD-NC vs. HC	1	71	Right precentral gyri	57	−12	24	−4.9068	0.005
**3. Slow5**
**Contrast**	**Cluster No.**	**Number of voxels**	**Main brain regions**	**Peak MNI coordinate**			***t* value**	***p* value**
				***X***	***Y***	***Z***
PD-MCI vs. HC	1	98	Putamen_R	21	0	3	−4.7292	0.01
	2	89	Putamen_L	−15	12	−3	−3.8054	0.01
PD-NC vs. HC	1	114	Putamen_R	30	−9	3	−4.4104	0.005
	2	80	Putamen_L	−27	−12	3	−4.6006	0.005

#### Groups Difference in fALFF Map

**Figure 4:**
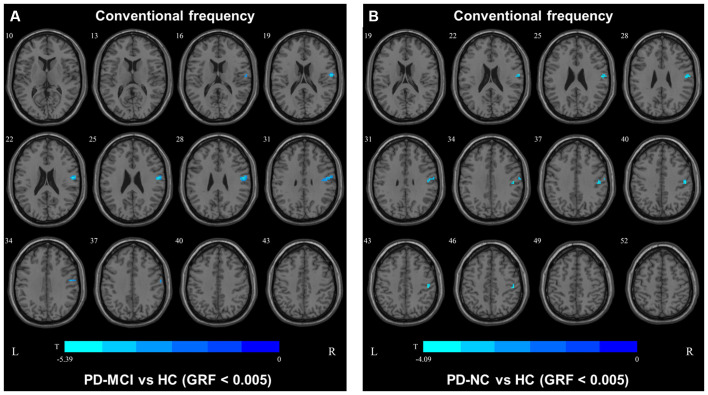
Fractional amplitude of low-frequency fluctuation (fALFF) map differences among the groups in conventional frequency band. The fALFF results were compared by two sample *t*-tests (GRF corrected, voxel *p* < 0.005, cluster *p* < 0.05). The color bar on the bottom indicates the statistical t value. The cold color indicates a significantly decreased fALFF in the comparison. Additional details regarding these regions with difference are shown in Table 3. **(A)** Decreased fALFF in PD-MCI patients between PD-MCI and HC. **(B)** Decreased fALFF in PD-NC patients between PD-NC and HC.

In the conventional frequency band, both PD-MCI and PD-NC patients showed decreased fALFF values in the area of the right precentral/postcentral gyri. However, no regions were significantly different between the PD-MCI and PD-NC groups (Figure 4, Table 3).

**Figure 5:**
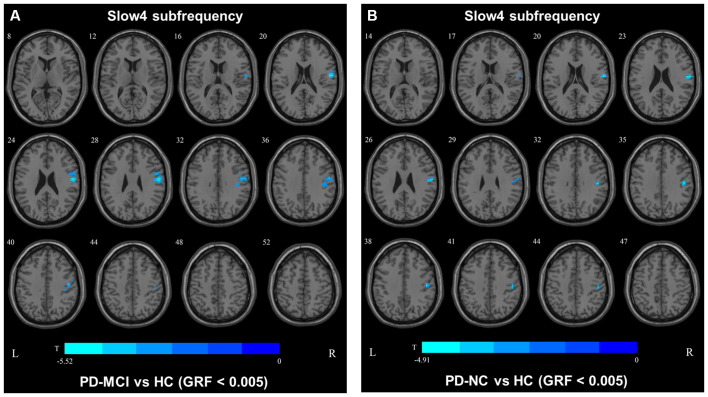
fALFF map differences among the groups in slow4 subfrequency band. The fALFF results were compared by two sample *t*-tests (GRF corrected, voxel *p* < 0.005, cluster *p* < 0.05). The color bar on the bottom indicates the statistical t value. The cold color indicates a significantly decreased fALFF in the comparison. Additional details regarding these regions with difference are shown in Table 3. **(A)** Decreased fALFF in PD-MCI patients between PD-MCI and HC. **(B)** Decreased fALFF in PD-NC patients between PD-NC and HC.

**Figure 6:**
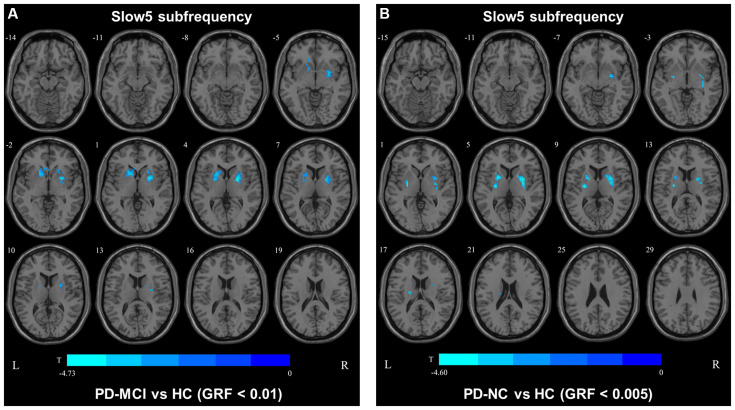
fALFF map differences among the groups in slow5 subfrequency band. The fALFF results were compared by two sample *t*-tests (PD-MCI vs. HC: GRF corrected, voxel *p* < 0.01, cluster *p* < 0.05; PD-MCI vs. HC: GRF corrected, voxel *p* < 0.005, cluster *p* < 0.05). The color bar on the bottom indicates the statistical *t*-value. The cold color indicates a significantly decreased fALFF in the comparison. Additional details regarding these regions with difference are shown in Table 3. **(A)** Decreased fALFF in PD-MCI patients between PD-MCI and HC. **(B)** Decreased fALFF in PD-NC patients between PD-NC and HC.

In the slow4 subfrequency band, the fALFF results were similar to those obtained in the conventional frequency band (Figure 5, Table 3). In the slow5 subfrequency band, the fALFF values in the left and right putamen were decreased in both PD-MCI and PD-NC patients (PD-NC: voxel *p* < 0.005, cluster *p* < 0.05, GRF correction, PD-MCI: voxel *p* < 0.01, cluster *p* < 0.05, GRF correction) compared with the HCs (Figure 6, Table 3).

#### Correlations Between Local Metrics and the Clinical Characteristics of PD-MCI

In the slow5 fALFF map, the fALFF values in the left and right putamens of PD-MCI patients were found to be correlated with the H-Y stage (left: *r* = −0.54, *p* = 0.0071; right: *r* = −0.47, *p* = 0.021), whereas only the left putamen was correlated with the MDS-UPDRS-III score (*r* = −0.50, *p* = 0.013), and the right putamen was correlated with the HAMA score (*r* = −0.43, *p* = 0.036).

In the conventional frequency DC map, the DC value of the left precentral area was correlated with the H-Y stage (*r* = −0.46, *p* = 0.024) and performance on the digit symbol test (*r* = 0.45, *p* = 0.029). In the slow4 subfrequency band, the DC value of the left precentral area was correlated with the HAMA score (*r* = −0.43, *p* = 0.034). In the slow5 subfrequency band, the DC value of the left middle temporal gyri were correlated with performance on the MoCA (*r* = 0.47, *p* = 0.019).

### Discussion

Most previous studies focused on the conventional frequency band of rs-fMRI and only ALFFs in PD patients with cognitive impairment (Hou et al., 2014; Wang et al., 2018; Tian et al., 2020). However, we herein compared the fALFF and DC values of PD-MCI patients in different subfrequency bands with those of PD-NC patients and HCs. We found that the DC values in the conventional and slow4 frequency bands could not distinguish PD-MCI from PD-NC; however, the DC values in the slow5 frequency band were significantly different between the PD-MCI and PD-NC groups. The DC value in the slow5 band was more effective and reliable for distinguishing PD-MCI from PD-NC than that in the conventional and slow4 frequency bands. Second, none of the fALFF values in the assessed frequency bands could distinguish PD-MCI from PD-NC, and both PD groups showed decreased fALFF values in the slow5 band in the bilateral putamen compared with those of the HCs.

#### Differences in the fALFF and DC Values in the Conventional Frequency Band Among the Groups

In this study, the fALFF values in the right precentral area were decreased in both the PD-MCI and PD-NC groups compared to the HCs, but the values did not differ between the PD groups. The right precentral area is a part of the sensorimotor network (SMN) that is correlated with disrupted functional integration in corticostriatal loops (Tessitore et al., 2014). Therefore, the decreased fALFF values in the precentral area suggested SMN dysfunction in the PD groups. Previous studies used ALFFs to investigate the disruption of neuronal activity in PD patients with cognitive decline (Gao et al., 2016; Wang et al., 2018), and their results differed from our fALFF results. To our knowledge, ALFF can be used to define the total power within the frequency range between 0.01 Hz and 0.1 Hz (Zang et al., 2007), and fALFF is defined as the total power within the low-frequency range (0.01–0.1 Hz) divided by the total power in the entire frequency range (Zou et al., 2008). A previous study suggested that while ALFF can be used to effectively detect low-frequency oscillation (LFO) fluctuations, the fluctuations extend over 0.1 Hz, especially near the major vessels (Zou et al., 2008). Therefore, the fALFF results obtained herein are more reliable than the ALFF results.

We also observed decreased DC values in the left precentral area, which is part of the SMN, in PD-MCI patients compared with HCs. However, the DC values in this area did not differ between the PD-MCI and PD-NC groups. Therefore, the DC value in the conventional frequency band did not distinguish PD-MCI from PD in our study. A previous study found that the DC value in the left fusiform gyri was decreased in PD-MCI subjects compared with PD-NC subjects (Li et al., 2021), which was inconsistent with our result. In our study, some participants were receiving levodopa treatment, and no significant differences were observed among the groups after correcting for the LEDD. Additionally, levodopa could normalize DC abnormalities in patients with PD (Zhong et al., 2019). Thus, the DC value in the conventional frequency band may not be capable of distinguishing PD-MCI patients treated with levodopa. The fALFF and DC values in the conventional frequency band could not effectively distinguish PD-MCI from PD-NC, whereas functional disruption was observed in the PD groups compared with the HCs. In our study, similar results were observed in the slow4 subfrequency band.

#### Differences in the fALFF and DC Values in the Slow5 Subfrequency Band Among the Groups

In the slow5 subfrequency band, the DC values were effective for distinguishing PD-MCI from PD-NC. The DC value in the left middle temporal region was increased in PD-MCI patients compared with PD-NC patients, and the DC value in this region was correlated with the performance of PD-MCI patients on the MoCA. The temporal lobe was initially thought to be correlated with cognitive impairment in subjects with neurodegenerative diseases. Previous studies revealed that abnormal brain network activity in the temporal gyri was negatively correlated with the cognitive performances of PD-MCI patients (Gao et al., 2016; Fiorenzato et al., 2019). A voxelwise meta-analysis of PD-MCI suggested robust GM volume decreases in the temporal area (Qin et al., 2020), and hypometabolism of the temporal gyri was found in PD patients with cognitive decline (Tard et al., 2015). Therefore, abnormal functional activity of the temporal gyri could be a symptom of cognitive impairment in patients with PD. We herein showed that the DC value in the slow5 band could distinguish PD-MCI from PD-NC. The DC value reflects the role and status of voxels in the brain network and represents the most local and directly quantifiable centrality measure(Buckner et al., 2009). Here, the DC value in the temporal gyri was increased, indicating the increased importance of this region in PD-MCI and the compensatory structure of brain functional activity in PD-MCI.

#### Discrepancy Between the Results of Different Subfrequency Bands

Although the fALFF values could not distinguish PD-MCI from PD-NC, both PD-MCI and PD-NC patients showed decreased fALFF values in the bilateral putamen compared with those of HCs in the slow5 band. The slow5 result differed from those of the conventional and slow4 frequency bands. Previous studies investigating different subfrequency bands in PD were concentrated on ALFFs and found differences in not only the basal ganglia and putamen but also some motor cortex regions (Hou et al., 2014; Tian et al., 2020; Wang et al., 2020). In our study, decreased fALFF values were restricted to the bilateral putamen and were correlated with the H-Y stage and motor performance, which is related to fALFF characteristics. fALFF is defined as the total power within the low-frequency range divided by the total power in the entire frequency range. As a normalized index of ALFF, fALFF can provide a more specific measure of low-frequency oscillatory phenomena (Zuo et al., 2010). While Zuo and his colleagues found that the LFO amplitude in the basal ganglia was more robust in the slow4 band than in the slow5 band (Zuo et al., 2010), our results suggest that the amplitude of the putamen in the slow5 band is likely influenced by PD. Many studies have suggested that the neural activities in different regions can be sensitively detected in different frequency bands (Buzsaki and Draguhn, 2004; Zuo et al., 2010), and putatively, a decreased fALFF value in the bilateral putamen in the slow5 band could be a characteristic of PD.

In our study, the slow5 frequency band, which ranged from 0.01–0.027 Hz, more effectively reflected functional disruption between PD and PD-MCI. Previous studies have reported that the slow4 frequency band provides more important information for understanding the pathogenesis of PD (Hou et al., 2014; Hu et al., 2020; Tian et al., 2020). However, all of these studies focused on ALFFs, which characterize spontaneous brain activity at each voxel. The slow4 subfrequency band is wider than the slow5 band and may contain more fluctuating signals that are not simply depictive of physiopathological states, but some signals could be without physiopathological significance. fALFF, as normalization of ALFF (Zou et al., 2008), can prevent irrelevant fluctuating signals in brain regions and reflect pathological functional disruptions in LFO regions, such as slow5. Additionally, a previous study reported that global topological properties were more dominant in lower frequency bands ranging from 0 to 0.015 Hz (Qian et al., 2015) which is contained in slow5. To our knowledge, the DC value reflects the degree of intrinsic FC across the brain, which represents a stable property of the cortical network (Buckner et al., 2009). Therefore, the fALFF and DC values in the slow5 band could reflect LFO changes.

## Limitations

There were some limitations in the current study. First, some of the patients in our study were diagnosed with *de novo* PD and thus underwent MRI scans in the absence of dopaminergic therapy. To reduce the impact of dopaminergic therapy on our study, we performed a similar analysis in which LEDD was included as the covariant, which yielded similar results. Second, the sample size was relatively small, which can result in false positive results in correlation analyses. Therefore, *p* < 0.005 was set as the voxel threshold to decrease the risk of false positives. Moreover, only a few regions were analyzed in our study due to the strict threshold and we also showed the result under the threshold *p* < 0.01. The accurate determination of thresholds for fMRI studies remains challenging and needs further exploration.

## Conclusion

Our study demonstrated differences in the fALFF and DC values in different frequency bands between PD-MCI and PD-NC patients. Both the PD-MCI and PD-NC patients had similar regional disruptions when compared with the HCs, but the conventional and slow4 frequency bands could not distinguish PD-MCI from PD-NC. Additionally, the DC value in the in slow5 band has potential as an effective local parameter to distinguish PD-MCI from PD-NC, as this value in the left middle temporal lobe differed between the PD-MCI and PD-NC patients and was correlated with MoCA performance. Moreover, the fALFF value in the slow5 band may demonstrate putamen activity disruption in PD. Our findings may be helpful for further revealing frequency-dependent resting functional disruption in PD-MCI.

## Data Availability Statement

The raw data supporting the conclusions of this article will be made available by the authors, without undue reservation.

## Ethics Statement

The studies involving human participants were reviewed and approved by Medical Ethics Committee of Guangdong Provincial People’s Hospital. The patients/participants provided their written informed consent to participate in this study.

## Author Contributions

Conception and organization of the research project: SR, CH, PZ, and YZ. Collection of clinical data of participant: CH, YL, XL, and RL. Statistical analysis: SH and YL. Review and critique of the statistical analysis: KN. Writing of the first draft: SR. Review and critique on the manuscript: LimW and LijW. Final approval for submission: YZ. All authors contributed to the article and approved the submitted version.

## Conflict of Interest

The authors declare that the research was conducted in the absence of any commercial or financial relationships that could be construed as a potential conflict of interest.

## Publisher’s Note

All claims expressed in this article are solely those of the authors and do not necessarily represent those of their affiliated organizations, or those of the publisher, the editors and the reviewers. Any product that may be evaluated in this article, or claim that may be made by its manufacturer, is not guaranteed or endorsed by the publisher.

## Footnotes

^1^ http://www.fil.ion.ucl.ac.uk/spm

^2^ http://www.restfmri.net/forum/RESTplusV1.2
